# Investigation on health economic burden of patients with mild cognitive impairmentand Alzheimer's disease in Qinghai area

**DOI:** 10.1002/alz70858_099632

**Published:** 2025-12-25

**Authors:** Luxuan Yan, Aiqin Zhu

**Affiliations:** ^1^ Department of Geriatrics, Qinghai People's Hospital, Xining, China; ^2^ Graduate School of Qinghai University, Xining, China; ^3^ Clinical Research Center for Geriatric Diseases of Qinghai, Xining, China; ^4^ Qinghai Provincial Hospital, Qinghai University, Xining, China

## Abstract

**Background:**

To explore the disease‐related economic burden of patients with amnestic mild cognitive impairment (aMCI) and Alzheimer 's disease (AD) at high altitude.

**Method:**

From September 2023 to May 2024,163 cases (age 72.70±11.32 years) of memory clinic in Qinghai Provincial People 's Hospital (average altitude<2300 meters) were collected, including 57 cases of MCI and 106 cases of AD (73 cases of mild to moderate dementia, 33 cases of severe dementia). The Chinese Alzheimer 's Disease Health Economics Burden Study Scale was used for evaluation.

**Result:**

Ordinal logistic regression analysis showed that male (OR=1.072) and female (OR=1.161), the increases of long‐term care frequency were risk factors for the aggravation of cognitive impairment in plateau area (*p* <0.05). Multiple linear regression analysis:low education level was a risk factor for the decrease of Mini‐Mental State Examination(MMSE) value (*p* <0.001);married and self‐care patients had a negative impact on MMSE value(b=‐3.049, ‐7.438), and a positive impact on Activities of Daily Living Assessment Scale (ADL) value (b=10.803, 18.73) and Clinical Dementia Rating(CDR) value (b=0.665,1.075)(*p* <0.01). ROC curve analysis showed that long‐term care frequency can predict the progress of cognitive impairment(AUC=0.955, *p* <0.001).

**Conclusion:**

Unmarried / divorced / widowed, low education level, more caregiving and long‐term care frequency increasing are risk factors for the aggravation of MCI and AD patients at high altitude.

